# Ac4C Enhances the Translation Efficiency of Vegfa mRNA and Mediates Central Sensitization in Spinal Dorsal Horn in Neuropathic Pain

**DOI:** 10.1002/advs.202303113

**Published:** 2023-10-25

**Authors:** Ting Xu, Jing Wang, Yan Wu, Jia‐Yan Wu, Wei‐Cheng Lu, Meng Liu, Su‐Bo Zhang, Dan Xie, Wen‐Jun Xin, Jing‐Dun Xie

**Affiliations:** ^1^ Neuroscience Program Zhongshan School of Medicine The Fifth Affiliated Hospital Guangdong Province Key Laboratory of Brain Function and Disease Department of Physiology and Pain Research Center Sun Yat‐Sen University Guangzhou 510080 China; ^2^ Department of Pain Management Henan Provincial People's Hospital Zhengzhou University Zhengzhou 450000 China; ^3^ Department of Anesthesiology The First Affiliated Hospital of Sun Yat‐Sen University Guangzhou Guangdong 510062 China; ^4^ State Key Laboratory of Oncology in Southern China Collaborative Innovation for Cancer Medicine Sun Yat‐Sen University Cancer Center Guangzhou 510060 China; ^5^ Department of Anesthesia and Pain Medicine Guangzhou First People's Hospital Guangzhou 510180 China

**Keywords:** ac4C modification, NAT10, neuropathic pain, spinal dorsal horn, translation efficiency, VEGFA

## Abstract

N4‐Acetylcytidine (ac4C), a highly conserved post‐transcriptional machinery with extensive existence for RNA modification, plays versatile roles in various cellular processes and functions. However, the molecular mechanism by which ac4C modification mediates neuropathic pain remains elusive. Here, it is found that the enhanced ac4C modification promotes the recruitment of polysome in Vegfa mRNA and strengthens the translation efficiency following SNI. Nerve injury increases the expression of NAT10 and the interaction between NAT10 and Vegfa mRNA in the dorsal horn neurons, and the gain and loss of NAT10 function further confirm that NAT10 is involved in the ac4C modification in Vegfa mRNA and pain behavior. Moreover, the ac4C‐mediated VEGFA upregulation contributes to the central sensitivity and neuropathic pain induced by SNI or AAV‐hSyn‐NAT10. Finally, SNI promotes the binding of HNRNPK in Vegfa mRNA and subsequently recruits the NAT10. The enhanced interaction between HNRNPK and NAT10 contributes to the ac4C modification of Vegfa mRNA and neuropathic pain. These findings suggest that the enhanced interaction between HNRNPK and Vegfa mRNA upregulates the ac4C level by recruiting NAT10 and contributes to the central sensitivity and neuropathic pain following SNI. Blocking this cascade may be a novel therapeutic approach in patients with neuropathic pain.

## Graphical abstract

Present study investigating the role of N4‐Acetylcytidine (ac4C) in neuropathic pain. Researchers discover that NAT10 mediates the increase in ac4C modification of Vegfa mRNA facilitating the binding of polysomes, leading to enhanced translation efficiency. The upregulated VEGFA contributes to neuropathic pain by inducing central sensitivity. These findings may suggest a novel therapeutic approach in patients with neuropathic pain.

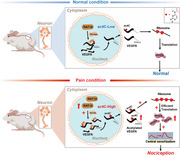

## 1 Introduction

Currently, most available treatment approaches for neuropathic pain usually lack efficacy or produce severe side effects, which may be attributable to the suboptimal understanding of the mechanism of regulating the expression of pain‐related molecules.^[^ ^1^ ^]^ Emerging evidence indicates that post‐transcriptional RNA modification, termed the epitranscriptome, participates in multiple neurobiological functions by regulating target gene expression.^[^ ^2^ ^]^ Understanding the post‐transcriptional mechanisms that regulate the expression of pain‐associated genes may pave a new potential avenue for neuropathic pain management.

Studies show that various RNA modifications, such as N6‐methyladenosine (m6A),^[^ ^3^ ^]^ 5‐methylcytosine (m5C),^[^ ^4^ ^]^ and ac4C,^[^ ^5^ ^]^ play a pivotal role in regulating RNA stability, subcellular localization, and translation.^[^ ^6^ ^]^ Notably, ac4C, a ubiquitous highly conserved post‐transcriptional machinery for RNA modification, exhibits versatile functions in the various cellular processes.^[^ ^7^ ^]^ For example, ac4C‐involved acetylation of RUNX2 prevented ovariectomy‐induced bone loss.^[^ ^8^ ^]^ In addition, ac4C acetylation was also involved in various nervous system diseases. It was reported that ac4C modification in long non‐coding RNAs might be associated with the occurrence and development of Alzheimer's Disease.^[^ ^9^ ^]^ Currently, it is unclear whether and how ac4C modification is involved in neuropathic pain in the spinal dorsal horn, which is the mainstay for nociceptive information relay and processing. N‐acetyltransferase 10 (NAT10), as a member of the GCN5‐related N‐acetyltransferases (GNAT) family of histone acetyltransferases, is the only known RNA ac4C “writer” protein in mammals.^[^ ^10^ ^]^ Several studies reported the involvement of NAT10 in multiple biological processes including aging and depression.^[^ ^11^ ^]^ However, no evidence exists to show the role and mechanism of NAT10‐mediated modification of mRNA acetylation in the spinal dorsal horn in neuropathic pain. In addition, peripheral nerve injury may regulate the expression of pain‐related genes at the post‐transcriptional level in the spinal dorsal horn.^[^ ^12^ ^]^ It is unknown which key pain‐related genes are regulated by ac4C modification and subsequently contribute to nerve injury‐induced neuropathic pain.

Vascular endothelial growth factors A (VEGFA) is an anti‐parallel homodimeric protein that belongs to the “Cys‐loop” superfamily of proteins. It is recognized as a key modulator of endothelial cell mitogenesis, vasculogenesis, and vascular permeability.^[^ ^13^ ^]^ Notably, VEGFA emerges in the CNS of primitive organisms without a well‐developed vasculature, which suggests that VEGFA might have additional functions rather than promoting blood vessel growth and maintenance.^[^ ^14^ ^]^ Indeed, early studies revealed a potential pro‐inflammatory activity of VEGFA, including upregulation of chemokines and adhesion molecules for leukocyte recruitment and trafficking.^[^ ^15^ ^]^ Furthermore, studies also showed a wide range of effects of VEGFA on neural cells during development and adulthood.^[^ ^16^ ^]^ For example, it promotes CNS perfusion, induces direct neurotrophic effects in normal and pathological conditions, and modulates the functionality of the blood‐brain barrier.^[^ ^17^ ^]^ Recent investigations that the VEGFA expression was significantly upregulated in the spinal cord following nerve injury and inhibition of VEGFA attenuated the nerve injury‐induced behavioral hypersensitivity,^[^ ^18^ ^]^ further implied that VEGFA was integral for the modulation of nociception and onset of neuropathic pain.^[^ ^19^ ^]^ Of note, whether and how ac4C‐mediated post‐transcriptional modifications are involved in nerve injury‐induced VEGFA upregulation remains largely unknown.

In this study, we showed for the first time that the increased ac4C level promoted the translation efficiency of Vegfa mRNA and facilitated the expression of VEGFA protein in the dorsal horn following SNI. Furthermore, the increased binding of HNRNPK on Vegfa mRNA enhanced NAT10 recruitment and upregulated the ac4C level on Vegfa mRNA. Consequently, upregulation of VEGFA induced the central sensitization in the dorsal horn and contributed to pain behavior induced by SNI.

## 2 Experimental Section

### 2.1 Animals

Male Sprague–Dawley rats weighing 200–250 g were obtained from the Institute of Experimental Animals of Sun Yat‐Sen University. The operated rats were individually housed and the naïve rats were housed in a cage in a group of three individuals. All rats were kept at 23 ± 2 °C and 50–60% humidity under a 12:12 h light/dark cycle and with ad libitum access to food and water. All experimental procedures were approved by the Institutional Animal Care and Use Committee (IACUC) at Sun Yat‐Sen University (China) and were conducted in accordance with the guidelines of the National Institutes of Health (NIH) on animal care and ethical guidelines.

### 2.2 Spared Nerve Injury and Behavioral Tests

The rats were under isoflurane (4%) anesthesia, and the tibial and common peroneal branches of the sciatic nerve were ligated and sectioned distal to the ligation with 2 mm of the distal nerve stump removed. In sham‐operated rats, the tibial and common peroneal branches of the sciatic nerve were identically exposed without ligation.

For measurement of mechanical allodynia, animals were placed in a plastic box on a metal mesh, and Von Frey filaments (Ugo Basile, Aesthesio) with different bending forces were applied alternately to the lateral part of the plantar surface of the hind paw. A nociceptive response was defined as a brisk paw withdrawal or paw flinching following Von Frey filament application. The 50% paw withdrawal threshold was calculated for each animal following a previously validated up‐down procedure.^[^ ^20^ ^]^

For the measurement of heat hyperalgesia, the animals were first acclimatized to the manipulations and behavioral apparatus (Anhui Zhenghua Biological Instrument Equipment Co., Ltd, ZH‐6C) for 2 days. During the formal experiment, the animals were gently dropped onto a heated plate (50 °C). The application of thermal stimuli to the animal's hind paws would elicit withdrawal responses, which were observed as paw licking. The latency or the time it took for the withdrawal response to occur, was recorded. Each experiment consisted of two latency measurements taken at 10 min intervals to obtain an average latency value.

### 2.3 Western Blotting

Proteins obtained from spinal dorsal horn tissues were separated by gel electrophoresis SDS‐PAGE and transferred to a PVDF membrane. The PVDF membrane was incubated with primary antibodies against NAT10 (Abcam; ab194297; 1:2000), VEGFA (Abcam; 214 424; 1:1000), HNRNPK (Proteintech; 11426‐1‐AP; 1:1000), GAPDH (CST; 2118; 1:1000) overnight at 4 °C. The blots were then incubated with HRP‐conjugated secondary antibodies. The immunostained bands were quantified using a computer‐assisted imaging analysis system (Image J).

### 2.4 Immunofluorescence

Rats were anesthetized, and cardiac perfusion was performed using 0.9% physiological saline, followed by 4% paraformaldehyde in PBS. Next, L4‐L6 spinal cord tissues were removed and post‐fixed for 30 min in the same fixative and then dehydrated with 30% sucrose. Cryostat sections (25 µm thick) were cut and blocked with 1% BSA for 1 h at room temperature and incubated with primary antibodies against NAT10 (Abcam; ab194297; 1:100), VEGFA (Abcam; 214 424; 1:100 and Proteintech; 66828‐1‐Ig; 1:100), HNRNPK (Abcam; ab3995; 1:200), NeuN (Millipore; MAB377; 1:200), GFAP (CST; 3670; 1:200), Iba1 (Abcam; ab5076; 1:200) at 4 °C overnight. The next day, the sections were incubated with Cy3, Alexa 488‐conjugated secondary antibody at room temperature for 1 h. The stained sections images were captured by a Nikon (ECLIPSE Ni‐E) microscope equipped with a 20X/0.75NA objective lens or by a Nikon confocal (C2) microscope equipped with a 100X/1.45NA objective lens or by a Zeiss LSM800 microscope equipped with a 63X/1.40NA objective lens.

### 2.5 Intrathecal and Intraspinal Injection

The remodelin (a potent inhibitor of NAT10), neutralizing antibody, or siRNA (RiboBio Co.Ltd) was used for intrathecal injection. The intrathecal injection was performed according to our previously described method.^[^ ^21^ ^]^ Laminectomy of the L5 vertebra was performed during anesthesia using sodium pentobarbital (50 mg kg^−1^, i.p.). After the dura was probed with an 8G needle, a polyethylene‐10 catheter was implanted into the L4–L6 intervertebral subarachnoid space through the L5/L6 intervertebral space, and the tip of the catheter was located between the levels of the L4–L6 spinal segments. After intrathecal implantation, the rats were allowed to recover for 5 days. Animals that exhibited hind limb paresis or paralysis were excluded from the study.

For intraspinal injection of the AAV (Obio Technology (Shanghai) Corp., Ltd.), the L4–L6 vertebrae were exposed, and the vertebral column was mounted in a stereotaxic frame. A slight laminectomy was performed, and the arachnoid was incised to expose the spinal cord. Injections were performed at a rate of 30 nL min^−1^ with glass micropipettes (tip diameter 30–40 µm) attached to a 10 µL syringe. The location of injections for the I‐III layer of the spinal cord was determined by the stereotaxic coordinates (ML, ±0.75 mm (the central blood vessel at point 0); DV, –0.25 mm). AAV viruses were injected at four different sites (150 nL of AAV was injected at each site) apart from the top and bottom for 1 mm. The glass micropipette was withdrawn 10 min after viral injection, and the incision was closed with stitches layer by layer.

### 2.6 RNA Extraction and Quantitative Real‐Time Polymerase Chain Reaction

Trizol was used to extract the total RNA of tissues. The reverse transcription was performed following the protocol of the polymerase chain reaction (PCR) production kit. The cycles of reaction included holding stage at 95 °C for 3 min and 40 times of thermal cycling with 10 s at 95 °C, 20 s at 58 °C, and 10 s at 72 °C. The ratio of mRNA expression in the dorsal horn tissues was analyzed by the 2^−ΔΔCT^ method. The primers used in the study are listed in **Table** **1**.

**Table 1:** Specific primer sequences for qPCR.

Gene	Primer	Sequence
Vegfa‐CDS (Rat)	Forward	5′‐ CACGACAGAAGGGGAGCAGAAAG ‐3′
	Reverse	5′‐ GGCACACAGGACGGCTTGAAG ‐3′
Vegfa‐3’UTR (Rat)	Forward	5′‐ GACCAATGAGGCACTGTCCG ‐3′
	Reverse	5′‐ GACCGCGACTGCAATACACA ‐3′
Srrm1‐CDS (Rat)	Forward	5′‐ GCCCAAGAAGACGCAAATCC ‐3′
	Reverse	5′‐ GTTCTGTGACGGGGAGATCG ‐3′
Schip1‐3’UTR (Rat)	Forward	5′‐ CATGCCTGCCTCGTAGTGAA ‐3′
	Reverse	5′‐ ACCATGTGGATCCGACAGAA ‐3′
S1pr1‐ CDS (Rat)	Forward	5′‐ GGGCCCCTCTCTTCATCCTA ‐3′
	Reverse	5′‐ CTGAGTTCAGCACAGCCAGA ‐3′
Timp3‐3’UTR (Rat)	Forward	5′‐ AGTCCGGGAGGCATTACTCT ‐3′
	Reverse	5′‐ CCTCTCCTCCTCAGTCCCAA ‐3′
β‐Actin (Rat)	Forward	5′‐TGGAAGAAGAGGCCTGGTAATG‐3′
	Reverse	5′‐GAAGGGTAAGCCACTCACACA‐3′

### 2.7 ac4C Dot Blot

RNA (1 µg) was heated to 65 °C for 5 min, then immediately placed on ice for 1 min, and then loaded onto Hybond‐N^+^ membranes (GE Amersham). Membranes were cross‐linked with 150 mJ cm^−2^ in a UV 254 nm. Then membranes were soaked with methylene blue for 15 min at room temperature and washed with double distilled water and PBST alternately until the spots were clear. The image was captured as an internal reference. Then the membranes were blocked with 5% non‐fat milk in PBST for 1 h at room temperature and incubated with an anti‐ac4C antibody (Abcam; ab252215; 1:1000) in PBST at 4 °C overnight. The membranes were next washed three times with PBST, probed with HRP‐conjugated secondary anti‐rabbit IgG in PBST at room temperature for 1 h, and washed three times with PBST. Chemiluminescent HRP substrate (Millipore) was used to visualize the dots. The immunostained bands were captured and quantified using a computer‐assisted imaging analysis system (ImageJ).

### 2.8 RNA‐Binding Protein Immunoprecipitation (RIP)

RIP experiments were performed using a Magna RIP RNA‐Binding Protein Immunoprecipitation Kit (Millipore; 17–700). Briefly, the spinal dorsal horn was collected and homogenized into a single‐cell suspension in ice‐cold PBS. After centrifugation, the pellet was resuspended in an equal volume of RIP lysis buffer. Magnetic beads were incubated with antibodies against NAT10 (Abcam; ab194297; 5 µg), HNRNPK (Proteintech; 11426‐1‐AP; 5 µg), or IgG at room temperature. The tissue lysates were then incubated with the bead‐antibody complexes overnight at 4 °C. After treatment with proteinase K, the immunoprecipitated RNAs were extracted and reverse transcribed. The abundance of Vegfa mRNA was detected by qPCR.

### 2.9 Acetylated RNA‐Binding Protein Immunoprecipitation (acRIP) and acRIP‐Seq

The total RNA was extracted using Trizol and fragmented by RNA fragmentation reagents (Thermo, AM8740). After saving 200 ng of the total RNA as input, the remaining RNAs (2 µg) were used for ac4C‐immunoprecipitation with ac4C antibody (Abcam, ab252215, 1:50) and Dynabeads Protein A (Thermo, 10006D), then eluted twice with elution buffer (5 mm Tris‐HCL pH 7.5, 1 mm EDTA pH 8.0, 0.05% SDS, 20 mg mL^−1^ Proteinase K). ac4C RIP RNAs were recovered by ethanol precipitation, and RNA concentration was measured with NanoDrop One (Thermo). Then the input RNA and ac4C RIP RNA were used as templates in qRT‐PCR. The primers for the acRIP‐RT‐qPCR are shown in **Table** **2**.

**Table 2:** The nucleotide sequences of siRNA.

Gene	Species	Sequence
NAT10	Rat	5′‐ AGGAATTCCAGGAGAAACA dTdT −3′
HNRNPK	Rat	5′‐ GGACGTGCACAACCTTATGAT dTdT −3′
VEGFA	Rat	5′‐ GCGGAUCAAACCUCACCAA dTdT‐ 3′

For ac4C RIP sequencing, the total polyadenylated RNA was isolated using Trizol from the spinal dorsal horn tissue of the rats with SNI or sham 14 days before. mRNA isolation, fragmentation, ac4C‐sequencing, and library preparation were performed by Guangzhou Epibiotek Co., Ltd. Ac4C‐seq data were analyzed according to protocols described before.^[^ ^5^ ^]^ The SNI and sham samples were aligned to the RefSeq database (rn6) genome assembly using Hisat2 aligner with default parameters. Peak calling was conducted based on exomePeak software and visualized by Metageneplot. Significant peaks were identified with FDR < 0.05. Identified peaks were further analyzed to screen sequence motifs using Homer. The differential acRIP‐seq genes between SNI and sham groups were identified with the criteria set as |log2(Fold Change)| > 1 and *p*‐value<0.05. Gene Set Enrichment Analysis (GSEA) based on the acRIP‐seq genes was performed by “clusterProfiler” R package.

### 2.10 Ribosome Profiling Sequencing (Ribo‐Seq) and RNA Sequencing

Isolation of the ribosome‐protected fraction, library construction, high‐throughput sequencing, and analysis were performed by Gene Denovo (Guangzhou, China). In brief, ribosome‐protected mRNA was obtained using MNase digestion and RNA purification.^[^ ^22^ ^]^ Three micrograms of ribosome‐protected mRNA was used to prepare a library with the NEBNext Small RNA Library Prep Set (New England Biolabs) in accordance with the manufacturer's instructions. After PCR amplification, the samples were used for quality control and deep sequencing with an Illumina HiSeq 2000. Raw reads were filtered and mapped to the rat ribosome RNA database using bowtie2. The reads on the aligned ribosomal RNA were removed, and the remaining were used for subsequent analysis. rn6 genome was used as a reference genome to determine the ribosome density and relative gene expression. Ribo‐seq data analysis was performed as previously described.^[^ ^23^ ^]^ Differential translated genes between groups were defined as |log2(FoldChange)| > 0.3 and *p*‐value<0.05.

For RNA‐seq, RNA extraction and polyadenylated RNA enrichment were performed by Gene Denovo (Guangzhou, China). In total, 5 µg of RNA was used to prepare for cDNA libraries using the cDNA‐PCR Sequencing Kit (SQK‐PCS109) according to the protocol provided by Oxford Nanopore Technologies (ONT). After PCR amplification, the samples were used for quality control and deep sequencing with an Illumina HiSeq 2000 followed by the computational analysis provided. Following adapter removal reads alignment and relative expression determination, RNA‐seq data processing was performed as instructed. Differentially expressed genes between groups were identified under parameters |(log2(FoldChange)| > 1 and *p*‐value<0.05) using the DESeq R‐package. Translational efficiency was defined as the ratio of translating mRNAs to total mRNAs as follows: TE = (FPKM in Ribo‐seq) / (FPKM in RNA‐seq). To determine the functional similarity with genes, the Friends analysis was applied using the GOSemSim package.^[^ ^24^ ^]^

### 2.11 Sucrose Gradient Fractionation

Polysome fractionations were performed as described previously (Kim et al., 2009). PC‐12 cells stimulated by glutamate or control cells (75 cm^2^ Cell Culture Flask) were treated with 100 mg mL^−1^ cycloheximide (ChX; GC34329; GLPBIO) for 10 min at 37 °C. Then, cells were harvested and 1 mL of cytoplasmic extract was layered onto 11 mL of 10%–50% sucrose gradient and centrifuged at 36 000 rpm in a Beckman SW‐41Ti rotor for 2.5 h at 4 °C. Gradients were fractionated and monitored at an absorbance of 254 nm (Brandel). Collected fractions were then analyzed by qPCR.

### 2.12 RNA Stability

To measure RNA stability in PC‐12 cells with or without glutamate treatment, actinomycin D (AcD; GC16866; GLPBIO) at 5 µg mL^−1^ was added to cells. After incubation for 1, 2, or 4 h, cells were collected, and RNA was isolated for RT‐qPCR.

### 2.13 RNA‐Pulldown LC/MS

The rats’ L4–L6 spinal dorsal horn tissues were quickly removed and homogenized into a single‐cell suspension in cold PBS (0.1 m). The suspension was cross‐linked with 3% formaldehyde at room temperature for 30 min, then quenched by the addition of 1.25 m glycine for 5 min. After centrifugation, the collected pellets were sonicated in an ice‐water bath. The biotinylated probes (**Table** **3**) for target mRNA were incubated with streptavidin beads for 30 min and mixed with tissue lysate at 37 °C overnight. For protein elution, beads were first collected on a magnetic stand. Then the beads were re‐suspended in biotin elution buffer and mixed at room temperature for 20 min at 65 °C for 10 min. The supernatants were incubated with 0.1% SDC and 10% TCA to precipitate proteins at 4 °C overnight. The next day, proteins were pelleted and then solubilized in 1x Laemmli sample buffer (Invitrogen) and boiled at 95 °C for 30 min with occasional mixing for reverse crosslinking. After tryptic digestion, the final protein samples were size‐separated in bis‐tris SDS PAGE gels (Invitrogen) for MS analysis.

**Table 3:** The nucleotide sequences of probe.

Probe name	Primer	Sequence
Vegfa (Rat)	Forward	5′‐ GCCATCAAGCTCTCTCCTCC −3′
	Reverse	5′‐ GGCCTCTTCTTCCACCACTG −3′

### 2.14 Field Potentials Recording In Vivo

The rats maintained all normal vital signs under anesthetizing with urethane (1.5 g kg^−1^, i.p.). The left sciatic nerve was isolated for putting a silver hook electrode. The lumbar segments 4 and 5 were exposed with a laminectomy. C‐fiber–evoked field potentials were recorded in ipsilateral lumbar enlargement with glass electrodes (impedance 1—2 mω) in response to sciatic nerve fiber stimulation. Data were digitized and collected by an A/D converter card (DT2821‐F‐16SE, Data Translation, MA) at a sampling rate of 10 kHz. Test stimuli were single square pulses (0.5 ms duration, in 1 min intervals), and stimulation strength was ≈1.5 times of the threshold for C‐fiber response. Long‐term potentiation (LTP) program was used to determine the amplitudes of C‐fiber‐evoked field potentials. Responses to five consecutive test stimuli were averaged for each experiment. The mean amplitudes of C‐fiber responses before drug or vehicle application served as baseline. Only 1 recording was conducted on each animal.

### 2.15 Whole‑Cell Patch Recordings

The recording chamber was continuously filled with pre‐warmed 33 °C artificial cerebrospinal fluid (ACSF) at a rate of 2 mL min^−1^. Pipettes (3–6 mω, ≈2 µm tip diameter) were drawn on a P‐2000G micropipette puller (Sutter Instruments, USA) using borosilicate glass (outer diameter: 1.5 mm, inner diameter: 0.86 mm). An EPC 10 amplifier (HEKA Elektronik, Germany) was used to record data. Patchmaster software (HEKA Elektronik) was used to deliver stimuli and acquire data. Electrophysiological data were processed and analyzed by Clampft 10.4 (Axon Instruments) and the mini‐analysis program (Synaptosoft Corp.).

For miniature excitatory postsynaptic current (mEPSC) recordings, the neurons in lamina I/II of L4‐6 spinal cord dorsal horn were voltage clamped at −70 mV, and mEPSCs were recorded after application of TTX (0.5 µm) and picrotoxin (100 µm). The pipette contained an internal solution containing (in mmol L^−1^): 135 K‐gluconate, 0.5 CaCl_2_, 2 MgCl_2_, 5 EGTA, 5 HEPES, and 5 Mg‐ATP, pH 7.3). To stain the recorded neurons, 0.5% biocytin (Sigma, USA) was also included in the internal solution. Current traces were recorded continuously for a period of 5 min after at least 5 min achieving whole‐cell con‐figuration and analyzed using MiniAnalysis program 6.0.7 (Synaptosoft Inc., Decatur, GA, USA).

Action potential (AP) recordings were made under current‐clamp conditions using a pipette filled with an internal solution containing (in mmol L^−1^): 135 K‐gluconate, 0.5 CaCl_2_, 2 MgCl_2_, 5 EGTA, 5 HEPES, 5 Mg‐ATP, pH 7.3). APs were induced by injecting currents every 10 s from −80 to 400 pA in step intervals of 20 pA throughout for 500 ms. Clampft (Axon Instruments) was used to analyze the relationship between frequency and injected current.

### 2.16 Co‐Immunoprecipitation (Co‐IP)

Co‐IP was conducted using a Pierce IP/Co‐IP Kit (Thermo, 88 804). Briefly, spinal dorsal horn tissues were excised quickly and placed in a lysis buffer. A Pierce Spin Column was placed in a microcentrifuge tube. After the addition of Amino Link Plus Coupling Resin and affinity‐purified antibody against NAT10 (Abcam; ab194297; 5 µg), HNRNPK (Proteintech; 11426‐1‐AP; 5 µg) or IgG, the complex was incubated on a rotator at room temperature for 120 min to ensure antibody immobilization. Tissue lysates were added to the appropriate resin columns and incubated with gentle rocking overnight at 4 °C. The spin columns were then centrifuged and placed in new collection tubes, elution buffer was added, and the flow‐through was collected by centrifugation. The immune complexes in the flow‐through were analyzed by western blotting using HNRNPK (Proteintech; 11426‐1‐AP; 1:1000), and NAT10 (Abcam; ab194297; 1:2000) antibodies. All co‐IP steps were performed at 4 °C unless otherwise indicated.

### 2.17 Statistics

SPSS 25.0 was used to analyze the data, and the results are shown as the mean ±SEM. The normally distributed data were analyzed using the two independent samples t‐test or one‐way ANOVA followed by Bonferroni or Dunnett's T3 post hoc test. When tests of normality were not satisfied, the non‐parametric test (Mann–Whitney test) was substituted. The data on pain behavior were analyzed using two‐way repeated‐measures ANOVA. The correlation was measured by Pearson correlation analysis. The criterion of statistical significance was 0.05. In order to minimize the number and suffering of animals as much as possible, the sample size was determined according to previous publications in behavioral and pertinent molecular studies. All measurements were taken from distinct samples.

## 3 Results

### 3.1 Nerve Injury Increases the ac4C Modification of Vegfa mRNA in the Spinal Dorsal Horn

**Figure 1:**
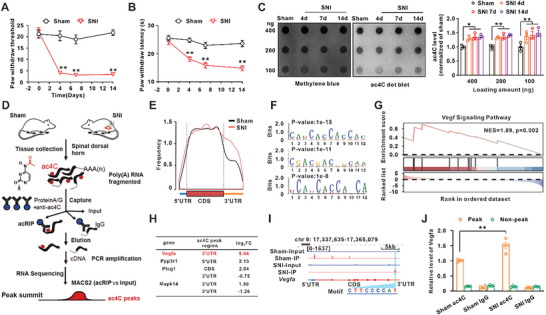
SNI induced the upregulation of ac4C modification of Vegfa mRNA. A) Paw withdrawal threshold of rats was significantly reduced on days 4, 7, and 14 following SNI (^**^ *p* < 0.01 vs corresponding sham group, *n* = 12 in each group). B) Heat hyperalgesia was induced on days 4, 7, and 14 after SNI (^**^ *p* < 0.01 vs corresponding sham group, *n* = 8 ‐ 9 in each group). C) Dot blot showed that the level of total RNA ac4C modification with different sample loading amounts increased on days 4, 7, and 14 following SNI in spinal dorsal horn tissue. Methylene blue staining was used as a loading control (^*^ *p* < 0.05, ^**^ *p* < 0.01 vs corresponding sham group, *n* = 3 in each group). D) Schematic of acRIP‐seq for mRNA of spinal dorsal horn tissue in rats. E) Metagene plot revealed the distribution of ac4C‐containing peaks across mRNA in sham and SNI groups. F) The motif of ac4C modification in the sham group and SNI group was identified by HOMER. G) VEGFA signaling pathway generated from GSEA. The top portion of the plot showed the enrichment score for the gene set of VEGF signaling pathway. The middle portion of the plot showed the members of the gene set of VEGF signaling pathway. The bottom portion showed the value of gene ranking in VEGF signaling pathway. The ranking value indicated an individual gene's correlation with the mechanical allodynia. H) The ac4C level in Vegfa mRNA exhibited the highest fold change following SNI in VEGF signaling pathway. I) Integrative Genomics Viewer (IGV) tracks displayed ac4C peak distributions across Vegfa mRNA from acRIP‐seq data. The ac4C modification sequence was CTTCCCCAT in the 3′UTR region in the SNI group. J) AcRIP‐qPCR showed that SNI significantly increased the ac4C level of Vegfa mRNA 3′UTR on day 14 (^**^ *p* < 0.01 vs corresponding sham group, *n* = 4 in each group).

Consistent with the peer's study,^[^ ^25^ ^]^ spared nerve injury (SNI) induced mechanical allodynia and heat hyperalgesia in the rats (**Figure** **1**A,B). To determine the potential role of RNA ac4C modification in neuropathic pain, we examined the changes in the global level of ac4C in the spinal dorsal horn after SNI in rats. In parallel with the time course of painful behavior, dot blot assay revealed that the ac4C level in total RNA was obviously increased on days 4, 7, and 14 following SNI relative to the sham group (Figure 1C). Spearman correlation analysis showed that the ac4C level of total RNA was reversely correlated with the mechanical paw withdrawal threshold following SNI (Figure S1A, Supporting Information). Similarly, incubation with glutamate (0.1 mg/ml, 48 h), which mimics a sustained glutamate release in the spinal dorsal horn induced by peripheral nerve injury,^[^ ^26^ ^]^ significantly increased the total RNA ac4C level in the PC‐12 cells (Figure S1B, Supporting Information). To identify the key targets for ac4C modification regulation in neuropathic pain, an ac4C‐RIP‐sequencing (acRIP‐seq) assay was conducted with mRNA isolated from dorsal horn tissue of the sham and SNI group (Figure 1D). The result showed that ac4C modification on mRNA was commonly presented in both sham and SNI groups (Figure 1E), and the motif sequence that underwent ac4C modification was “‘CXXCXXCXX”’ (C represents cytidine, X represents any ribonucleotide) (Figure 1F). The peaks immunopurified by ac4C were mapped to 2962 genes in the sham group and 1870 genes in the SNI group (Table S1, Supporting Information). Compared to the sham group, Gene Set Enrichment Analysis (GSEA) showed that ten signaling pathways met significance with a p‐value of less than 0.05 (Figure S1C, Supporting Information), among which the VEGF signaling pathway had the highest normalized enrichment score (NES) (Figure 1G; Figure S1C, Supporting Information). Moreover, in VEGF signaling pathway, the most prominent change was the increase of Vegfa mRNA ac4C level (Figure 1H), which was enriched near the 3′UTR (Chr9:17354697‐17354996) containing the classic ac4C motif, following SNI (Figure 1I). Furthermore, we confirmed the ac4C level in Vegfa mRNA 3′UTR region by using the acRIP‐qPCR assay in the spinal dorsal horn. The mRNA was precipitated with an antibody against N4‐Acetylcytidine (ac4C), and then subjected to PCR to amplify the target sequence with specific primers. The results showed that the ac4C level of Vegfa mRNA was significantly increased on day 14 following SNI (Figure 1J), and the time course of ac4C modification in Vegfa mRNA was paralleled with that of painful behavior (Figure 1A,B; Figure S1D, Supporting Information). These results suggested that the ac4C level in Vegfa mRNA was significantly increased in the spinal dorsal horn following SNI.

### 3.2 ac4c Modification Improves the Translation Efficiency of Vegfa mRNA

**Figure 2:**
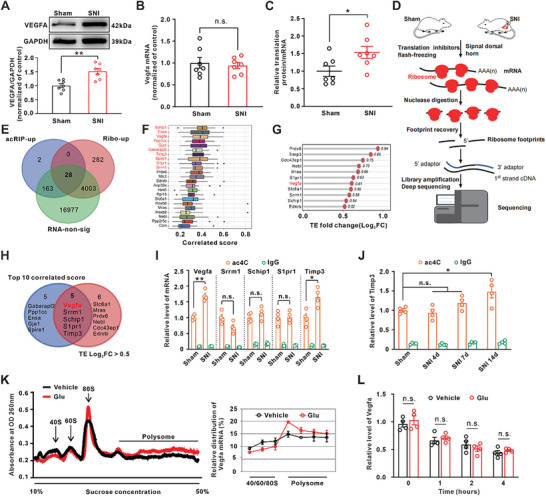
Ac4C modification improved the translation efficiency of Vegfa mRNA in neuropathic pain. A) Western blot showed the upregulation of VEGFA on day 14 following SNI (^**^ *p* < 0.01 vs corresponding sham group, *n* = 7 in each group). B) qPCR analysis showed the relative expression of Vegfa mRNA in the spinal dorsal horn on day 14 following SNI (*n* = 7 in each group). C) Compared with the sham group, the ratio of VEGFA protein to its mRNA was significantly increased following SNI (^*^ *p* < 0.05 vs corresponding sham group, *n* = 7 in each group). D) Schematic diagram of Ribo‐seq for spinal dorsal horn tissue in the rats. E) Venn diagram showed the integration results of acRIP‐seq, Ribo‐seq, and RNA‐seq. F) Friends analysis showed the key genes associated with neuropathic pain and highly correlated with each other. Vegfa showed a strong correlation with other genes. G) By calculating the ratio of ribosome number (Ribo‐up)/unchanged mRNA (RNA‐non‐sig), the genes with increased translation efficiency (Log_2_ fold change>0.5) were obtained. H) Venn diagram showed the intersection obtained from Friends analysis (F) and genes with increased translation efficiency (G). I) AcRIP‐qPCR was performed to explore the ac4C level of five mRNA on day 14 after SNI (^*^ *p* < 0.05, ^**^ *p* < 0.01 vs corresponding sham group, *n* = 4 in each group). J) The ac4C level of Timp3 mRNA was examined at different time points following SNI (^*^ *p* < 0.05 vs corresponding sham group, *n* = 4 in each group). K) The representation of polysome profiling of PC‐12 cells following glutamate incubation, and relative levels of Vegfa mRNA in each ribosome fraction were quantified (right side). L) The actinomycin D assay showed that glutamate incubation did not change the level of Vegfa mRNA at different time points when compared with the vehicle *n* = 4 in each group.

Studies showed that the ac4C modification regulated the transcript expression by controlling the translation efficiency and the stability of mRNA.^[^ ^5^, ^27^ ^]^ Here, we found that the expression of VEGFA protein, but not mRNA, was significantly increased in the spinal dorsal horn on day 14 following SNI (**Figure** **2**A,B), and the upregulation of the protein/mRNA ratio suggested an enhanced translation efficiency (TE) of Vegfa mRNA (Figure 2C). Since translation efficiency can generally be defined by ribosome density on each mRNA molecule.^[^ ^28^ ^]^ To determine whether ac4C modification increases VEGFA expression by regulating translation efficiency, we performed the Ribo‐seq of dorsal horn tissue (Figure 2D). The results showed that the ribosome enrichment in 4313 genes was significantly increased following SNI when compared with the sham group (Table S2, Supporting Information). Next, we integrated the results of Ribo‐seq (the upregulated gene), acRIP‐seq (the upregulated gene), and RNA‐seq (the unchanged gene), then subsequently obtained 28 genes. Hence, the upregulation of these 28 genes was potentially mediated by the enhanced translation efficiency regulated by ac4C following SNI (Figure 2E). Furthermore, the Friends Analysis showed that Vegfa had a strong correlation with the other genes (Figure 2F) and exhibited a significant increase in translation efficiency (Log_2_FC > 0.5) (Figure 2G). Based on the above two analysis methods, we obtained five genes including Vegfa, Srrm1, Schip1, S1pr1, and Timp3 (Figure 2H). AcRIP‐qPCR further showed that the ac4C level of Vegfa and Timp3 mRNA, but not Srrm1, Schip1, and S1pr1, was significantly increased on day 14 after SNI (Figure 2I). Furthermore, we examined the ac4C level of Vegfa and Timp3 mRNA at different time points following SNI. The results showed that the ac4C level of Vegfa mRNA, but not Timp3 mRNA, was significantly increased (Figure 2J; Figure S1D, Supporting Information), and the time course of the Vegfa mRNA ac4C level upregulation was consistent with that of neuropathic pain following SNI (Figure 1A,B). To determine whether the upregulation of VEFGA may be attributable to the enhanced translation efficiency mediated by ac4C modification, we measured the density of ribosome on the Vefga mRNA fractions using sucrose gradient centrifugation in PC‐12 cells. The qPCR results showed that glutamate treatment significantly increased the polysome density (> 80S) on the Vegfa mRNA (Figure 2K). In addition, we performed the actinomycin D assay to assess the stability of the mRNA in PC‐12 cells and found that glutamate incubation did not change the level of Vegfa mRNA when compared with the vehicle group at different time points (Figure 2L). These data suggested that the increase in Vefga mRNA translation efficiency rather than the stability mediated the VEGFA protein upregulation.

### 3.3 NAT10 Mediated ac4C Modification of Vegfa mRNA and Neuropathic Pain Following SNI

**Figure 3:**
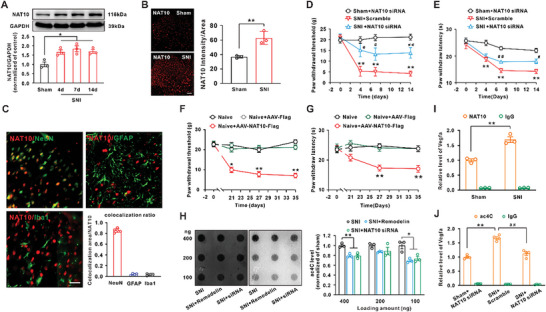
NAT10 contributed to the upregulation of ac4C modification in Vegfa mRNA and neuropathic pain following SNI. A) The expression of NAT10 protein was significantly increased on days 4, 7, and 14 following SNI (^*^ *p* < 0.05 vs sham group, *n* = 3 in each group). B) Immunofluorescence staining showed the NAT10 integrated density in the spinal dorsal horn on day 14 after sham and SNI (scale bar = 100 µm, *n* = 3 in each group). C) Colocalization of NAT10‐positive cells with NeuN‐positive cells (a marker for neurons), GFAP‐positive cells (a marker for astrocytes), or Iba1‐positive cells (a marker for microglia) in spinal dorsal horn were quantified (scale bar  =  25 µm, *n* = 4 or 5 in each group). D) Intrathecal injection of NAT10 siRNA alleviated the mechanical allodynia induced by SNI (^**^ *p* < 0.01 vs corresponding sham group, ^#^ *p* < 0.05, ^##^ *p* < 0.01 vs corresponding SNI group, *n* = 12 in each group). E) Intrathecal injection of NAT10 siRNA alleviated the heat hyperalgesia induced by SNI (^**^ *p* < 0.01 vs corresponding sham group, ^#^ *p* < 0.05, ^##^ *p* < 0.01 vs corresponding SNI group, *n* = 8 in each group). F) Twenty one days after intraspinal injection of AAV‐NAT10‐Flag, the withdrawal threshold was significantly reduced in naïve rats (^*^ *p* < 0.05, ^**^ *p* < 0.01 vs corresponding AAV‐Flag group, *n* = 10 in each group). G) Overexpression of NAT10 by injecting AAV‐NAT10‐Flag decreased the withdrawal latency in naïve rats (^**^ *p* < 0.01 vs corresponding AAV‐Flag group, *n* = 8 in each group). H) Application of remodelin or NAT10 siRNA inhibited the ac4C upregulation in the spinal dorsal horn on day 14 following SNI (^*^ *p* < 0.05, ^**^ *p* < 0.01 vs corresponding sham group, *n* = 3 in each group). I) The interaction between NAT10 and Vegfa mRNA ac4C sites was increased on day 14 following SNI (^**^ *p* < 0.01 vs corresponding sham group, *n* = 3 in each group). J) Application of NAT10 siRNA inhibited the increase of ac4C level at Vegfa mRNA ac4C sites induced by SNI on day 14 (^**^ *p* < 0.01 vs corresponding sham group, ^##^ *p* < 0.01 vs corresponding SNI, *n* = 4 in each group).

To explore the mechanism underlying ac4C modification of Vegfa mRNA in the spinal dorsal horn of rats following SNI, we first measured the expression of NAT10, the only enzyme known to mediate the acetylation of mRNA. The expression of NAT10 protein was significantly upregulated on days 4, 7, and 14 following SNI (**Figure** **3**A). Similarly, glutamate treatment also increased the NAT10 expression in PC‐12 cells (Figure S2A, Supporting Information). The increased fluorescence intensity confirmed the NAT10 upregulation on day 14 following SNI (Figure 3B), and the NAT10 protein was mainly expressed in the neurons (NeuN‐positive cells), though sparingly in the astrocytes (GFAP‐positive cells) or microglia (Iba1‐positive cells) (Figure 3C). We measured the expression of NAT10 at different levels of the spinal cord from T8 to S3; the highest expression was found in laminae I‐III of L4‐L6 (Figure S2B, Supporting Information). Behavioral tests showed that knockdown of NAT10 by using NAT10 siRNA (i.t.), which significantly suppressed the expression of NAT10 mRNA and protein (Figure S2C–E, Supporting Information), alleviated the mechanical allodynia and heat hyperalgesia in the rats following SNI (Figure 3D,E). Similarly, continuous intrathecal injection of remodelin (a NAT10 activity inhibitor) significantly attenuated the mechanical allodynia induced by SNI (Figure S2F,G, Supporting Information). Moreover, we overexpressed the NAT10 in the spinal dorsal horn by intraspinal injecting AAV‐NAT10‐Flag, in which obvious Flag‐positive signal in neurons and up‐regulation of NAT10 protein indicated the high transfected efficiency of AAV‐NAT10‐FLAG (Figure S3A,B, Supporting Information). Behavioral results showed that overexpression of NAT10 significantly decreased the withdrawal threshold and withdrawal latency in naïve rats (Figure 3F,G). Furthermore, we explored the role of NAT10 in the CCI model, and found that CCI significantly increased the NAT10 expression and the application of remodelin alleviated CCI‐induced mechanical allodynia (Figure S3C,D, Supporting Information). These results demonstrated that NAT10 upregulation served as a sufficient and necessary condition for neuropathic pain induced by SNI.

Dot blot results further showed that the application of remodelin (i.t) or NAT10 siRNA (i.t.) inhibited the upregulation of total RNA ac4C modification induced by SNI on day 14 in the spinal dorsal horn (Figure 3H). Similarly, in PC‐12 cells, preincubation of remodelin or NAT10 siRNA also prevented the increase of total RNA ac4C modification induced by glutamate incubation (Figure S3E, Supporting Information). Importantly, following SNI, the Vegfa mRNA site that underwent ac4C modification bound a large amount of NAT10 (Figure 3I). In addition, intrathecal injection of NAT10 siRNA or remodelin inhibited the increase of ac4C modification of Vegfa mRNA (Figure 3J; Figure S3F, Supporting Information). These results suggested that NAT10 positively regulated the ac4C modification of Vegfa mRNA in the spinal dorsal horn following SNI.

### 3.4 VEGFA Upregulation Is Involved in Central Sensitivity and Neuropathic Pain Following SNI

**Figure 4:**
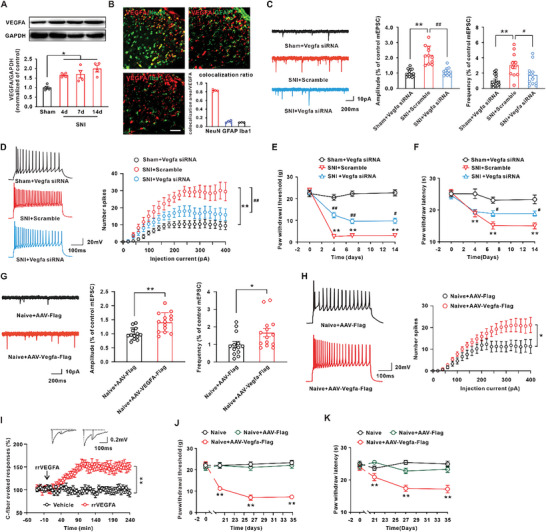
The VEGFA upregulation contributed to the central sensitivity and neuropathic pain following SNI. A) The level of VEGFA was significantly increased on days 4, 7, and 14 after SNI (^*^ *p* < 0.05 vs corresponding sham group, *n* = 4 in each group). B) Colocalization of VEGFA‐positive cells with NeuN‐positive cells (a marker for neurons), GFAP‐positive cells (a marker for astrocytes), or Iba1‐positive cells (a marker for microglia) in spinal dorsal horn was quantified (scale bar = 50 µm, *n* = 3 or 4 in each group). C) Vegfa siRNA treatment ameliorated the increase in the amplitude and frequency of mEPSCs on day 14 induced by SNI (^**^ *p* < 0.01 vs the sham group, ^#^ *p* < 0.05, ^##^ *p* < 0.01 vs the corresponding SNI group, *n* = 11–14 cells from 7–9 rats in each group). D) The number of depolarization‐induced neuronal firing was reduced in Vegfa siRNA‐treated rats following SNI (^**^ *p* < 0.01 vs the sham group, ^##^ *p* < 0.01 vs the corresponding SNI group, *n* = 11–13 cells from 7–8 rats in each group). E,F) Intrathecal injection of Vegfa siRNA alleviated the SNI‐induced mechanical allodynia and heat hyperalgesia (^**^ *p* < 0.01 vs corresponding sham group, ^#^ *p* < 0.05, ^##^ *p* < 0.01 vs corresponding SNI group, *n* = 7–10 in each group). G) Intraspinal injection of recombinant AAV‐hySn‐Vegfa‐Flag increased the amplitude and frequency of mEPSCs (^*^ *p* < 0.05, ^**^ *p* < 0.01 vs the AAV‐Flag group, *n* = 13‐14 cells from 8 rats in each group). H) Overexpression of VEGFA increased the number of depolarization‐induced neuronal firing in naïve rats (^*^ *p* < 0.05 vs the AAV‐Flag group, *n* = 12‐15 cells from 8 rats in each group). I) Local application of VEGFA onto the spinal dorsal horn significantly enhanced the C‐fiber–evoked field potential in naive rats. The trace at the top was recorded before (left) and 210 min after (right) VEGFA application. (^**^ *p* < 0.01 relative to the vehicle group, *n* = 4 in each group). J,K) Overexpression of VEGFA by intraspinal injection of AAV‐Vegfa significantly decreased the mechanical withdrawal threshold and heat hyperalgesia in the naïve rats (^**^ *p* < 0.01 vs corresponding naive group, *n* = 8–10 in each group).

Next, we explored the role of increased VEGFA in neuropathic pain. The western blot results showed that the VEGFA protein was significantly upregulated on days 4, 7, and 14 following SNI in the spinal dorsal horn (**Figure** **4**A). In addition, the time course of VEGFA protein upregulation was consistent with that of neuropathic pain and positively correlated with ac4C modification of Vegfa mRNA (Figure 1A; Figure S4A, Supporting Information). Immunofluorescence staining revealed that the VEGFA immunoreactivity in neurons (NeuN positive cells) was significantly higher in comparison with that in astrocytes (GFAP positive cells) or microglia (Iba1 positive cells) (Figure 4B). Electrophysiological studies showed that the amplitude and frequency of miniature excitatory postsynaptic currents (mEPSCs) and the number of depolarization‐induced neuronal firing were significantly increased in NK1R‐positive neurons in the spinal dorsal horn on days 14 after SNI (Figure 4C,D; Figure S4B, Supporting Information). Inhibition of VEGFA by injection of Vegfa siRNA ameliorated the increase in the amplitude and frequency of mEPSCs and the depolarization‐induced neuronal firing on days 14 after SNI (Figure 4C,D). A subsequent behavioral test revealed that knockdown of VEGFA significantly elevated the withdrawal threshold and the withdrawal latency (Figure 4E,F). Intrathecal injection of anti‐VEGFA neutralizing antibody (0.2 mg kg^−1^) also alleviated the SNI‐induced mechanical allodynia (Figure S4C, Supporting Information). Importantly, we also noted that VEGFA siRNA injection per se did not change the behavioral sensitivity in the sham rats (Figure 4E,F). To further test whether VEGFA in the dorsal horn contributes to neuropathic pain, AAV‐hSyn‐Vegfa‐Flag was intraspinally injected into the L4–L6 spinal cord to overexpress VEGFA. Twenty‐one days after virus injection, the significant Flag‐positive signal in neurons and the increased VEGFA levels in the dorsal horn suggested a high specificity and efficiency of transfection (Figure S4D,E, Supporting Information). Compared with the AAV‐Flag group, overexpression of VEGFA significantly increased the amplitude and frequency of mEPSCs and depolarization‐induced neuronal firing (Figure 4G,H). In addition, local application of rat recombination VEGFA (rrVEGFA) (100 ng mL^−1^) induced long‐term potential of C‐fiber‐evoked field potential in naïve rats (Figure 4I). Importantly, intraspinal injection of AAV‐Vegfa‐Flag obviously induced mechanical allodynia and heat hyperalgesia in naïve rats (Figure 4J,K). These results suggested that the spinal cord VEGFA contributed to the central sensitization and behavioral hypersensitivity.

### 3.5 NAT10‐Mediated VEGFA Upregulation Contributed to Neuropathic Pain Induced by SNI

**Figure 5:**
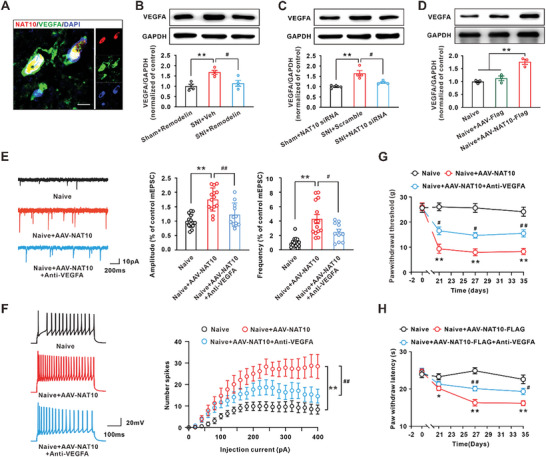
NAT10 mediated VEGFA upregulation and contributed to central hypersensitivity and neuropathic pain following SNI. A) The confocal image showed that NAT10 was expressed on the nucleus of VEGFA‐positive cells in the spinal dorsal horn (scale bar = 10 µm, *n* = 3). B) Intrathecal injection of remodelin inhibited the VEGFA upregulation induced by SNI (^**^ *p* < 0.01 relative to the sham group, ^#^ *p* < 0.05 relative to the corresponding SNI group, *n* = 4 in each group). C) The level of VEGFA protein in the spinal dorsal horn was decreased after intrathecal injection of NAT10 siRNA in SNI rats (^**^ *p* < 0.01 relative to the sham group; ^#^ *p* < 0.05 relative to the corresponding SNI group, *n* = 4 in each group). D) Intraspinal injection of AAV‐hySn‐NAT10‐Flag increased the level of VEGFA protein in the spinal dorsal horn in naïve rats (^**^ *p* < 0.01 relative to the corresponding naive group, *n* = 3 in each group). E) Application of anti‐VEGFA neutralizing antibody inhibited the increase in the amplitude and frequency of mEPSCs in the AAV‐hySn‐NAT10‐Flag rats (^**^ *p* < 0.01 vs the naïve group, ^#^ *p* < 0.05, ^##^ *p* < 0.01 vs the AAV‐NAT10 group, *n* = 11‐15 cells from 8–9 rats in each group). F) Application of anti‐VEGFA neutralizing antibody inhibited the increase in the number of depolarization‐induced neuronal firing induced by the AAV‐hySn‐NAT10‐Flag treatment (^**^ *p* < 0.01 vs the naive group, ^##^ *p* < 0.01 vs the AAV‐NAT10 group, *n* = 10‐14 cells from 7–8 rats in each group). G,H) Intrathecal injection of anti‐VEGFA neutralizing antibody also alleviated the mechanical allodynia and heat hyperalgesia induced by AAV‐hySn‐NAT10‐Flag in rats (^*^ *p* < 0.05, ^**^ *p* < 0.01 vs naive group, ^#^ *p* < 0.05, ^##^ *p* < 0.01 vs AAV‐NAT10 group, *n* = 7–9 in each group).

We further examined whether VEGFA upregulation was mediated by NAT10 in SNI‐induced neuropathic pain. The results showed that NAT10 was colocalized with VEGFA in the spinal dorsal horn (**Figure** **5**A), and inhibition of NAT10 activity by intrathecal injection of remodelin prevented the Vegfa mRNA ac4C modification as well as VEGFA upregulation on day 14 following SNI (Figure 5B; Figure S3F, Supporting Information). Knockdown of NAT10 by injection of NAT10 siRNA inhibited ac4C modification of Vegfa mRNA and VEGFA upregulation on day 14 following SNI (Figures 3J and 5C). Moreover, NAT10 overexpression by intraspinal injection of the AAV‐hySn‐NAT10‐Flag significantly increased the VEGFA levels (Figure 5D). Importantly, electrophysiological studies showed that the amplitude and frequency of mEPSCs and depolarization‐induced neuronal firing were significantly increased on day 21 after AAV‐hySn‐NAT10‐Flag injection (Figure 5E,F). Furthermore, intrathecal injection of anti‐VEGFA neutralizing antibody inhibited the increase in the amplitude and frequency of mEPSCs and the depolarization‐induced neuronal firing in the AAV‐hySn‐NAT10‐Flag rats (Figure 5E,F). In addition, intrathecal injection of anti‐VEGFA neutralizing antibody also alleviated the mechanical allodynia and heat hyperalgesia induced by AAV‐hySn‐NAT10‐Flag (Figure 5G,H). These data indicated that the NAT10‐mediated VEGFA upregulation contributed to central sensitivity and neuropathic pain following SNI.

### 3.6 HNRNPK Contributes to ac4C Modification in Vegfa mRNA by Recruiting NAT10

**Figure 6:**
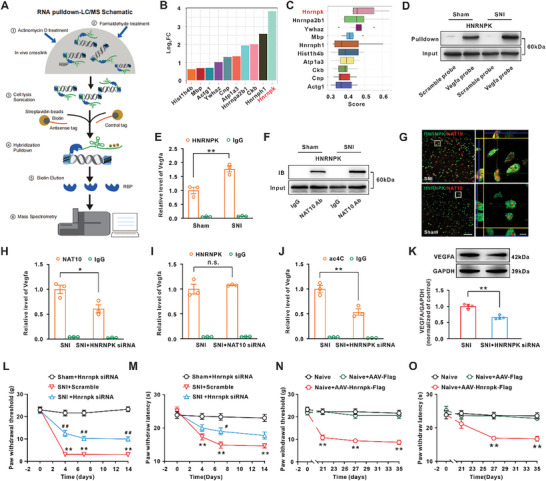
The enhanced interaction between HNRNPK and Vegfa mRNA contributed to the ac4C modification in rats by recruiting NAT10. A) Schematic diagram of RNA pulldown‐LC/MS/MS with Vegfa probe for spinal dorsal horn tissue in rats. B) Results from RNA pulldown‐LC/MS/MS assay showed an interaction between HNRNPK and Vegfa mRNA. C) Friends Analysis showed that the interaction of HNRNPK with other proteins was the most obvious. D) RNA pulldown‐WB assay showed an enhanced interaction between Vegfa mRNA and HNRNPK following SNI (*n* = 3 in each group). E) The Vegfa mRNA level immunoprecipitated by the HNRNPK antibody was significantly increased (*n* = 3 in each group). F) Co‐immunoprecipitation results showed that the interaction between NAT10 and HNRNPK was significantly increased in the spinal dorsal horn following SNI (*n* = 3 in each group). G) High‐resolution images showed that the binding of NAT10 to HNRNPK was significantly upregulated following SNI (scale bar = 50 µm in the left and scale bar = 5 µm in the right, *n* = 3 in each group). H) Intrathecal injection of HNRNPK siRNA inhibited the enhanced recruitment of NAT10 on Vefga mRNA in SNI rats (^*^ *p* < 0.05 relative to the corresponding SNI group, *n* = 4 in each group). I) Intrathecal injection of NAT10 siRNA did not change the interaction of HNRNPK and Vegfa mRNA in SNI rats (*n* = 3 in each group). J) HNRNPK siRNA treatment prevented the increase of Vegfa mRNA ac4C modification induced by SNI (^**^ *p* < 0.05 relative to the corresponding SNI group, *n* = 3 in each group). K) Knockdown of HNRNPK by siRNA inhibited the SNI‐induced VEGFA upregulation in the spinal dorsal horn of rats (^**^ *p* < 0.05 relative to the corresponding SNI group, *n* = 4 in each group). L,M) Knockdown of HNRNPK by siRNA alleviated the SNI‐induced mechanical allodynia and heat hyperalgesia (^**^ *p* < 0.01 vs corresponding sham group, ^#^ *p* < 0.05, ^##^ *p* < 0.01 vs corresponding SNI group, *n* = 10 in each group for mechanical allodynia test and *n* = 8 in each group for heat hyperalgesia test). N,O) Overexpression of HNRNPK by intraspinal injection of AAV‐Hnrnpk significantly decreased the mechanical withdrawal threshold and heat hyperalgesia in the naïve rats (^**^ *p* < 0.01 relative to the corresponding naïve group, *n* = 10 in each group for mechanical allodynia test and *n* = 8 in each group for heat hyperalgesia test).

To clarify the mechanism underlying the NAT10‐mediated ac4C modification in Vegfa mRNA, we performed the RNA pulldown‐LC/MS/MS assay using the Vegfa probe, which targets the ac4C site of Vegfa mRNA (**Figure** **6**A). The results revealed that 166 proteins might interact with the Vegfa mRNA (Table S3, Supporting Information). Among these proteins, HNRNPK and Vegfa mRNA had an obvious interaction (Figure 6B), and HNRNPK showed a strong correlation with other proteins (Figure 6C). RNA‐pulldown assay showed that the HNRNPK content immunopurified by the Vegfa probe was significantly increased following SNI (Figure 6D). RIP results further confirmed an enhanced interaction between HNRNPK and Vegfa mRNA ac4C site following SNI (Figure 6E). We then further determined whether HNRNPK was involved in the ac4C modification of Vegfa mRNA in neuropathic pain. Co‐immunoprecipitation results showed that the HNRNPK content immunoprecipitated by NAT10 antibody was significantly increased in the SNI group (Figure 6F). Consistently, the NAT10 content in the immunocomplex precipitated by the HNRNPK antibody was also significantly increased following SNI (Figure S4F, Supporting Information). In addition, high‐resolution images suggested that HNRNPK was colocalized with the NAT10 in spinal dorsal horn neurons and the colocalization significantly increased at day 14 after SNI (Figure 6G). RIP results showed that application of HNRNPK siRNA (i.t) inhibited the enhanced binding of NAT10 to Vegfa mRNA (Figure 6H), while NAT10 siRNA did not affect the interaction of HNRNPK and Vegfa mRNA (Figure 6I). Importantly, HNRNPK siRNA (i.t) also prevented the increased levels of Vegfa mRNA ac4C and VEGFA protein induced by SNI (Figure 6J,K). Finally, inhibition of HNRNPK by injection of siRNA elevated the withdrawal threshold and the withdrawal latency in SNI rats (Figure 6L,M), and overexpression of HNRNPK by intraspinal injection of AAV‐Hnrnpk‐Flag obviously induced the mechanical allodynia and heat hyperalgesia in naïve rats (Figure 6N,O). These results suggested that the enhanced interaction between HNRNPK and Vegfa mRNA upregulated the ac4C level by recruiting NAT10 and contributed to the neuropathic pain following SNI.

## 4 Discussion

Understanding the post‐transcriptional mechanism underlying the expression of pain‐related genes may provide novel potential targets for the optimal management of neuropathic pain. In the present study, we identified for the first time the mechanism and function of ac4C modification in Vegfa mRNA in neuropathic pain. Firstly, we observed that the increased ac4C modification of Vegfa mRNA primarily occurred in the specific site (CXXCXXCXX) of the 3′‐UTR region following SNI. The upregulated ac4C modification promoted the recruitment of polysome on Vegfa mRNA and enhanced the translation efficiency, rather than the stability, of Vegfa mRNA. Moreover, the translation efficiency‐mediated VEGFA upregulation enhanced the spinal central sensitivity and subsequently contributed to the mechanical allodynia and heat hyperalgesia following SNI. Nerve injury increased the expression of NAT10, the only enzyme for regulating RNA ac4C in mammals, and the interaction between NAT10 and Vegfa mRNA in the dorsal horn neurons. Further assays with the gain and loss of NAT10 function revealed that NAT10 modified the ac4C level of Vegfa and VEGFA expression and regulated behavioral hypersensitivity in neuropathic pain. Notably, the application of an anti‐VEGFA antibody prevented central sensitivity and neuropathic pain in the NAT10 overexpression rats. Finally, NAT10‐mediated upregulation of ac4C modification in Vegfa mRNA required the involvement of HNRNPK, since suppression of HNRNPK by siRNA reduced the NAT10 binding and ac4C level on Vegfa mRNA, and subsequently inhibited the VEGFA upregulation and neuropathic pain induced by SNI. Taken together, our findings indicated that NAT10‐regulated ac4C modification enhanced the translation efficiency of Vegfa mRNA, which consequently induced Vegfa upregulation and neuropathic pain following SNI.

Studies showed that ac4C, as the first acetylation modification identified on mRNA, was related to the development and prognosis of many diseases.^[^ ^29^ ^]^ Here, we found that the ac4C level was significantly increased in PC‐12 cells with glutamate incubation and the spinal dorsal horn following nerve injury. Further, the acRIP‐seq assay showed 2962 genes in the sham group and 1870 genes in the SNI group existed ac4C modification on mRNA. Although a recent study showed the role of ac4C‐mediated SYT9 expression in neuropathic pain in DRG,^[^ ^30^ ^]^ whether and how ac4C in the spinal dorsal horn was involved in neuropathic pain remained unclear. Here, the GSEA analysis showed that various genes enriched with ac4C were primarily distributed in ten signaling pathways, in which the Vegf signaling pathway with the highest normalized enrichment score (NES) was reportedly related to nerve injury.^[^ ^31^ ^]^ Moreover, GSEA analysis also confirmed that Vegfa may be a hub gene that was regulated by ac4C modification in the spinal dorsal horn in neuropathic pain. Ac4C, as a widespread marker of human mRNAs, enhanced transcript stability and translation efficiency.^[^ ^32^ ^]^ Evidence also showed that the ribosome density per mRNA molecule reflected the efficiency of gene translation.^[^ ^28^, ^33^ ^]^ Utilizing the ribo‐seq method, we found that SNI increased the ribosome enrichment on the Vegfa mRNA, and polysome fractionations analysis further confirmed the increased polysome abundance on the Vegfa mRNA in PC‐12 cells following glutamate incubation. Notably, western blot and qPCR revealed that the expression of Vegfa protein, but not mRNA, was significantly upregulated in the dorsal horn tissue following SNI. The results from the actinomycin D assay also showed that glutamate incubation did not affect the stability of Vegfa mRNA in PC‐12 cells. These results suggested that ac4C‐mediated enhancement of translational efficiency contributed to the VEGFA upregulation induced by nerve injury. Inhibition of VEGFA by siRNA or VEGFA neutralizing antibody alleviated the increase in the amplitude and frequency of mEPSCs, the number of depolarization‐induced neuronal firing, and neuropathic pain. Furthermore, local overexpression of VEFGA by intraspinal injection of AAV‐hySn‐VEGFA induced the central sensitization in the spinal dorsal horn and the painful behavior in naïve rats. In previous studies, VEGFA was widely recognized to regulate blood vessel function and integrity.^[^ ^34^ ^]^ Recently, accumulating studies have found that alterations in VEGFA expression and signaling profiles are strongly associated with a number of pathologies including diabetes, cancers, and macular degeneration.^[^ ^35^ ^]^ Research showed that the PI3K/AKT pathway is required for spinal central sensitization and the application of VEGFA mediates the activation of AKT.^[^ ^36^ ^]^ In addition, evidence reveals that the VEGFA/VEGFR pathway is involved in chemotherapy‐induced neuropathic pain.^[^ ^37^ ^]^ Our results showed that the ac4C‐regulated VEGFA upregulation was also involved in the central sensitization in dorsal horn neurons and neuropathic pain induced by nerve injury. So, it is possible that the upregulated VEGFA increases central sensitivity by activating pain‐related pathways. Of note, evidence showed that the molecular mechanism underlying chronic pain may be different between male and female animals. Further studies are in demand to clarify the potential sex dimorphism regarding the involvement of spinal VEGFA signaling in the development of neuropathic pain. Taken together, these results were also consistent with the studies that VEGFA acted upon the sensory neurons by inducing sensory neuron axonal outgrowth and survival to regulate the neuropathic pain.^[^ ^18^, ^38^ ^]^

With the development of epigenetic concepts and technology, evidence showed that ac4C, as a conservative chemical RNA modification catalyzed by the acetyltransferase NAT10,^[^ ^29^ ^]^ was involved in the pathogenesis of various diseases including metabolic diseases and cancer.^[^ ^39^ ^]^ By NAT10‐specific RIP sequencing method, a recent study showed that ac4C modification was significantly increased in cancer‐induced bone pain in rats.^[^ ^8^ ^]^ However, whether the spinal NAT10 participated in the neuropathic pain induced by SNI is not clarified. Our data revealed that SNI increased the ac4C level and NAT10 expression in dorsal horn neurons, and suppression of NAT10 significantly alleviated the upregulation of ac4C modification and the neuropathic pain induced by SNI. In addition, overexpression of NAT10 directly induced mechanical allodynia and heat hyperalgesia in naïve rats. These data demonstrated for the first time that NAT10 mediated the ac4C modification in the spinal dorsal horn and contributed to the process of neuropathic pain. In addition, SNI increased the NAT10 recruitment on the Vegfa mRNA 3′UTR, and inhibition of NAT10 by either remodelin or siRNA remarkably inhibited the SNI‐induced ac4C modification on Vegfa mRNA. Importantly, the gain and loss of NTA10 function respectively altered the expression of VEGFA in the spinal dorsal horn, and the application of VEGFA neutralizing antibody inhibited the enhanced central sensitivity and painful behavior in the AAV‐hySn‐NAT10 rats. These results suggested that NAT10 directly regulated ac4C modification on Vegfa mRNA to contribute to neuropathic pain.

Evidence shows that heterogeneous nuclear ribonucleoprotein K (HNRNPK) is a DNA/RNA‐binding protein and regulates a wide range of biological processes and disease pathogenesis.^[^ ^40^ ^]^ To clarify the mechanism underlying the NAT10‐mediated Vegfa mRNA ac4C modification, we performed the RNA pulldown‐LC/MS/MS using a Vegfa probe. The results showed that SNI increased the binding of HNRNPK on the Vegfa mRNA and the interaction between HNRNPK and NAT10 as well. Intrathecal injection of HNRNPK siRNA inhibited the enrichment of NAT10 and ac4C modification on Vegfa mRNA induced by SNI. While application of NAT10 siRNA did not affect the interaction of HNRNPK and Vegfa mRNA. Multiple pathophysiologic processes and mechanisms are involved in the development and maintenance of neuropathic pain. Structurally, the development and maintenance of neuropathic pain involve the peripheral nociceptor, central nervous system, and sympathetic nervous system. Mechanically, many molecules and signaling pathways contribute to neuropathic pain.^[^ ^41^ ^]^ In the present study, the knockdown of NAT10 or VEGFA using siRNA did not result in a complete reversal of neuropathic pain, which highlighted the involvement of other molecular mechanisms and pathways in neuropathic pain. Nonetheless, these data indicated that the enhanced interaction between HNRNPK and Vegfa mRNA upregulated the ac4C level by recruiting NAT10 and contributed to neuropathic pain following SNI.

**Figure 7:**
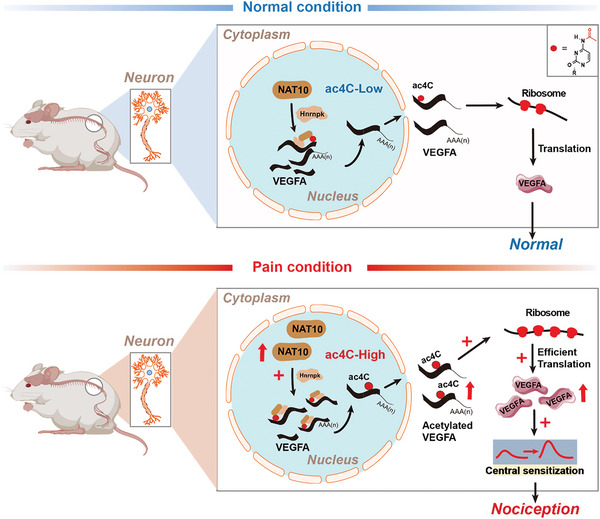
Summary graph for the hypothesis: the mechanism involving NAT10‐mediated VEGFA upregulation contributed to neuropathic pain following SNI.

Taken together, our results illustrated an ac4C‐mediated post‐transcriptional mechanism through which NAT10 regulated Vegfa expression in dorsal horn neurons and contributed to central sensitization and pain behavior induced by nerve injury. These findings identified a series of novel targets for the development of effective treatment for neuropathic pain induced by nerve injury (**Figure** **7**).

## Conflict of Interest

The authors declare no conflict of interest.

## Author Contributions

T.X., J.W., and Y.W. contributed equally to this work. J.‐D.X., W.‐J.X., and D.X. designed the experiments. T.X., J.W., Y.W., J.‐Y.W., and S.‐B.Z. performed the experiments. W.‐C.L., Y.W., and M.L. acquired the data. T.X., J.W., and J.‐D.X. analyzed the data. J.‐D.X., W.‐J.X., and D.X. wrote the manuscript.

## Supporting information

Supporting Information

Supplemental Table 1

Supplemental Table 2

Supplemental Table 3

## Data Availability

The data that support the findings of this study are available from the corresponding author upon reasonable request.
