# Supplementary material for: Ac4C Enhances the Translation Efficiency of Vegfa mRNA and Mediates Central Sensitization in Spinal Dorsal Horn in Neuropathic Pain
Source: Adv Sci (Weinh). 2023 Oct 25;10(35):2303113. doi: 10.1002/advs.202303113 (PMC10724395; doi:10.1002/advs.202303113)
Supplement: Supplementary file 1 — Supporting Information [file ADVS-10-2303113-s003.pdf]

ADVANCED SCIENCE
Open Access

Supporting Information

for *Adv. Sci.*, DOI 10.1002/advs.202303113

Ac4C Enhances the Translation Efficiency of Vegfa mRNA and Mediates Central Sensitization in Spinal Dorsal Horn in Neuropathic Pain

Ting Xu, Jing Wang, Yan Wu, Jia-Yan Wu, Wei-Cheng Lu, Meng Liu, Su-Bo Zhang, Dan Xie, Wen-Jun Xin* and Jing-Dun Xie**

Ac4C enhances the translation efficiency of Vegfa mRNA and mediates central sensitization in spinal dorsal horn in neuropathic pain

Ting Xu[1, #], Jing Wang[1, 2, #], Yan Wu[3, #], Jia-Yan Wu[1], Wei-Cheng Lu[4], Meng Liu[5], Su-Bo Zhang[4], Dan Xie[4, *], Wen-Jun Xin[1, *], Jing-Dun Xie[4, *]

[1] Neuroscience Program, Zhongshan School of Medicine, The Fifth Affiliated Hospital, Guangdong Province Key Laboratory of Brain Function and Disease, Department of Physiology and Pain Research Center, Sun Yat-sen University, Guangzhou, 510080, China

[2] Department of Pain Management, Henan Provincial People’s Hospital, Zhengzhou University, Zhengzhou, 450000, China

[3] Department of Anesthesiology, The First Affiliated Hospital of Sun Yat-sen University, Guangzhou 510062, Guangdong, China

[4] State Key Laboratory of Oncology in Southern China, Collaborative Innovation for Cancer Medicine, Sun Yat-sen University Cancer Center, Guangzhou, 510060, China

[5] Department of Anesthesia and Pain Medicine, Guangzhou First People's Hospital, Guangzhou 510180, China

#These authors contributed equally to this work

*** Corresponding Authors:**

Jing-Dun Xie, Department of Anesthesiology, Sun Yat-sen University Cancer Center, 651 Dongfeng East road, Guangzhou, China. E-mail: xiejd6@mail.sysu.edu.cn; Wen-Jun Xin, Zhongshan School of Medicine, Sun Yat-sen University, No.74 Zhongshan Rd. 2, Guangzhou, China. E-mail: xinwj@mail.sysu.edu.cn; Dan Xie, State Key Laboratory of Oncology in South China, Collaborative Innovation Center for Cancer Medicine, Sun Yat-sen University Cancer Center, 651 Dongfeng East road, Guangzhou, China. E-mail: xiedan@sysucc.org.cn

Supporting information

Supplementary Figures

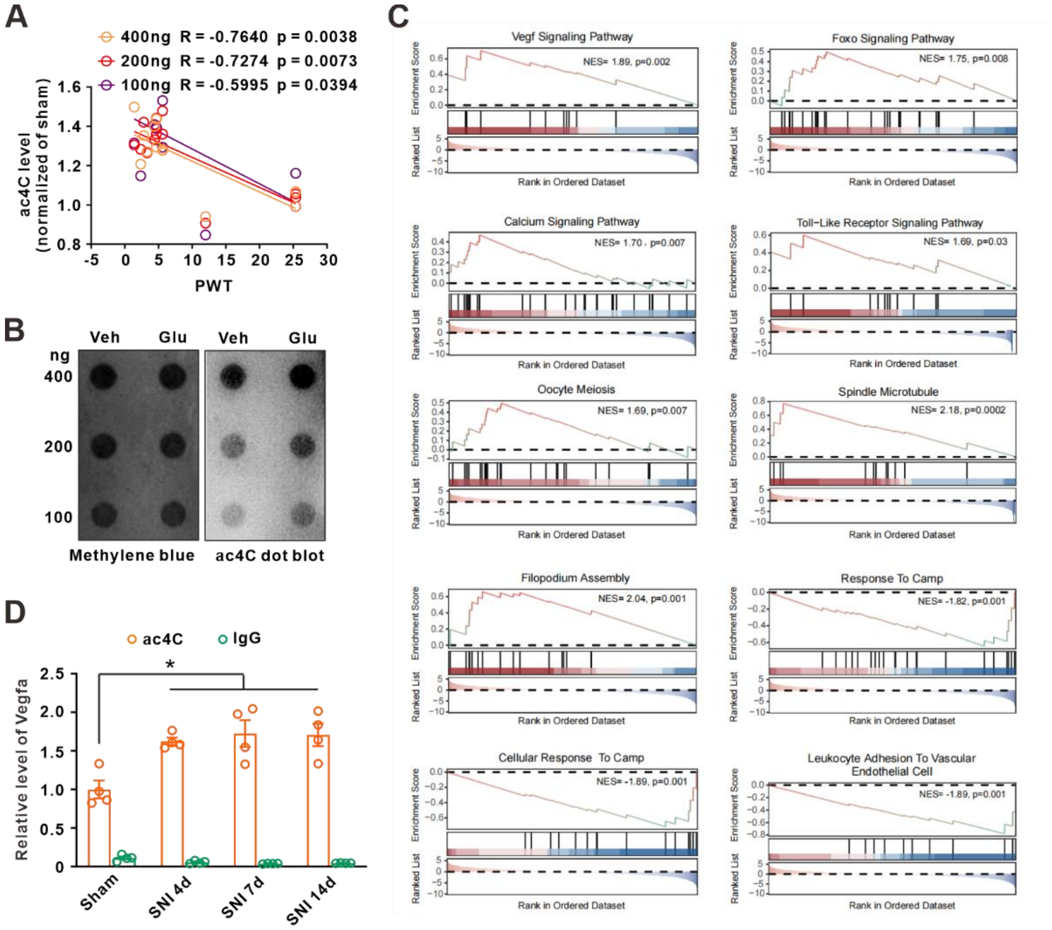

Supplementary Fig. 1 *(A) Correlation between the level of RNA ac4C modification and mechanical paw withdrawal threshold (PWT) in the SNI rats (n = 12 in each group). (B) Incubation of glutamate for 48 h increased the ac4C abundance in the total RNA in PC-12 cells (n = 3 in each group). (C) GSEA showed ten upregulated signaling pathways related to the neuropathic pain. (D) The level of Vegfa mRNA ac4C was increased on days 4, 7 and 14 following SNI (*P < 0.05 versus corresponding sham group, n = 4 in each group).*

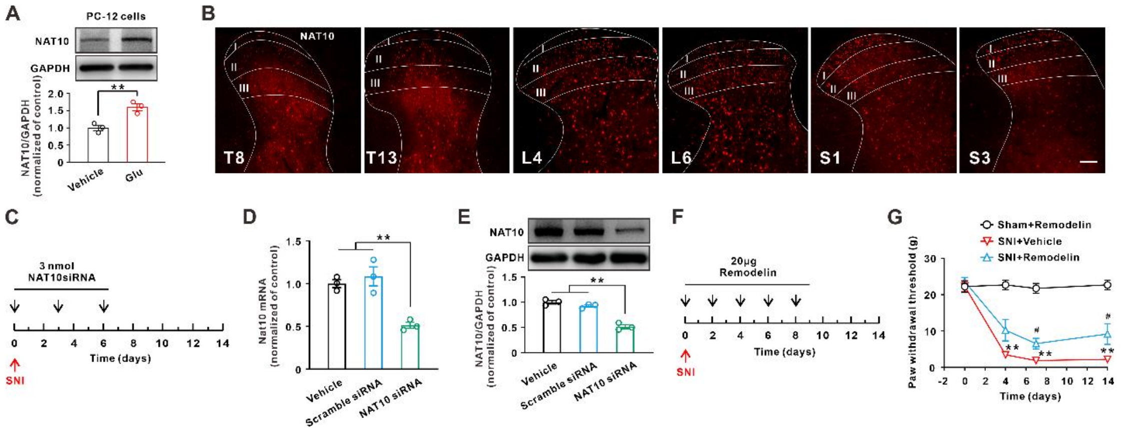

Supplementary Fig. 2 *(A) Glutamate incubation for 48 h increased the expression of NAT10 protein*

*in PC-12 cells (**p<0.01 versus sham group, n = 3 in each group). (B) The expression of NAT10 at different levels of the spinal cord from T8 to S3 in rats (scale bar = 100 μm, n = 3 in each group). (C) Scheme of experiment for intrathecal injection of NAT10 siRNA. (D and E) NAT10 siRNA (i.t.) significantly decreased the expression of NAT10 mRNA and protein (**p<0.01 versus the correspondence control group, n = 3 in each group). (F) Experimental schedule for intrathecal injection of remodelin and measurement of pain behavior. (G) Intrathecal injection of NAT10 activity inhibitor remodelin significantly attenuated the mechanical allodynia induced by SNI (** p<0.01 versus corresponding sham group, # p<0.05 versus corresponding SNI group, n = 8 in each group).*

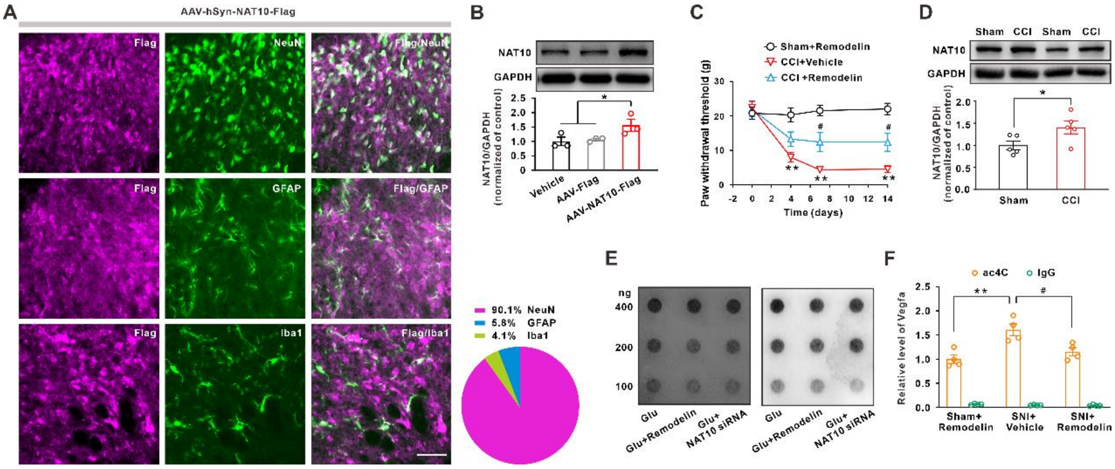

Supplementary Fig. 3 *(A) Flag immunoreactivity was analyzed in the spinal dorsal horn in rats. Colocalization with NeuN-positive neurons, GFAP-positive astrocyte and Iba-1 positive microglia was evaluated and quantified following intraspinal injection of AAV-NAT10-Flag (scale bar = 50 μm, n = 3 in each group). (B) The expression of NAT10 was increased in spinal dorsal horn following intraspinal injection of AAV-NAT10-FLAG (*P < 0.05 vs the correspondence AAV-Flag group, n=3 in each group). (C) Intrathecal injection of NAT10 activity inhibitor remodelin significantly attenuated the mechanical allodynia induced by CCI (** p<0.01 versus corresponding sham group, # p<0.05 versus corresponding CCI group, n = 8 in each group). (D) The expression of NAT10 protein was significantly increased on day 14 following CCI (*p<0.05 versus sham group, n = 5 in each group). (E) Inhibition of NAT10 by remodelin or siRNA prevented the increased level of total ac4C modification induced by glutamate in PC-12 cells (n = 3 in each group). (F) Intrathecal injection of remodelin inhibited the increase of ac4C level at Vegfa mRNA ac4C sites induced by SNI on day 14 (**P < 0.01 versus corresponding sham group, #P < 0.05 versus corresponding SNI, n = 4 in each group).*

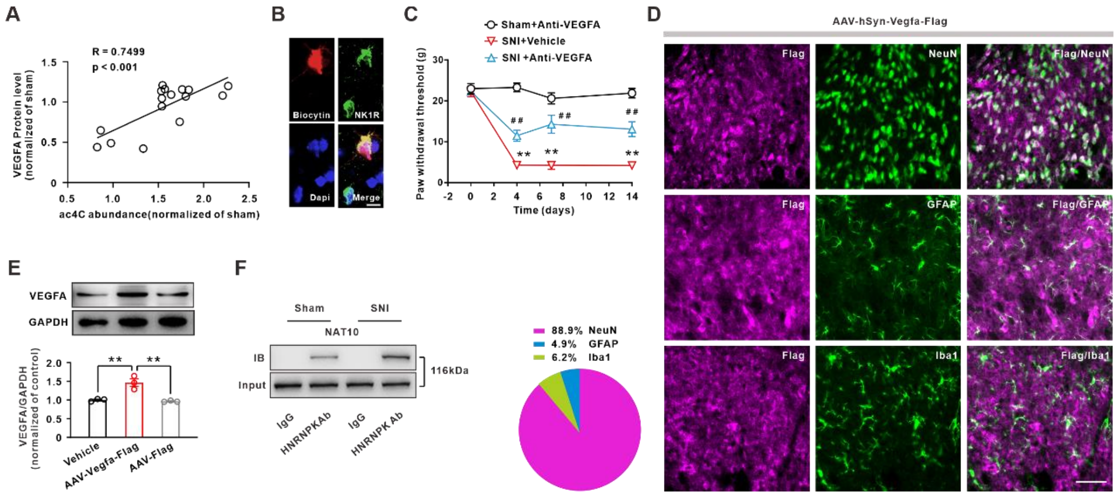

Supplementary Fig. 4 *(A) Correlation between the VEGFA protein and the level of RNA ac4C in the SNI rats (n = 12). (B) The co-localization of biocytin and NK1R to confirm NK1R+ neurons for electrophysiological studies in spinal cord slices (scale bar=10 μm). (C) Intrathecal injection of anti-VEGFA neutralizing antibody alleviated the SNI-induced mechanical allodynia (**p<0.01 versus correspondence sham group, ## p<0.01 versus corresponding SNI group, n = 10 in each group). (D) Flag immunoreactivity was analyzed in the spinal dorsal horn in rats. Colocalization with NeuN-positive neurons, GFAP-positive astrocyte and Iba-1 positive microglia was evaluated and quantified (scale bar=50 μm, n = 3 in each group). (E) Intraspinal injection of AAV-Vegfa-Flag obviously induced the expression of VEGFA protein (**p<0.01 versus the correspondence control group, n = 3 in each group). (F) Co-immunoprecipitation results showed that the NAT10 content in the immunocomplex precipitated by HNRNPK antibody was significantly increased following SNI (n = 3 in each group).*
